# Relating proton pumps with gap junctions: colocalization of ductin, the channel-forming subunit c of V-ATPase, with subunit a and with innexins 2 and 3 during *Drosophila* oogenesis

**DOI:** 10.1186/s12861-016-0124-y

**Published:** 2016-07-13

**Authors:** Julia Lautemann, Johannes Bohrmann

**Affiliations:** Institut für Biologie II, RWTH Aachen University, Abt. Zoologie und Humanbiologie, Worringerweg 3, 52056 Aachen, Germany

**Keywords:** *Drosophila melanogaster*, Oogenesis, Bioelectricity, Cell communication, Pattern formation, Proton pump, Gap junction

## Abstract

**Background:**

Ion-transport mechanisms and gap junctions are known to cooperate in creating bioelectric phenomena, like pH gradients, voltage gradients and ion fluxes within single cells, tissues, organs, and whole organisms. Such phenomena have been shown to play regulatory roles in a variety of developmental and regenerative processes. Using *Drosophila* oogenesis as a model system, we aim at characterizing in detail the mechanisms underlying bioelectric phenomena in order to reveal their regulatory functions. We, therefore, investigated the stage-specific distribution patterns of V-ATPase components in relation to gap-junction proteins.

**Results:**

We analysed the localization of the V-ATPase components ductin (subunit c) and subunit a, and the gap-junction components innexins 2 and 3, especially in polar cells, border cells, stalk cells and centripetally migrating cells. These types of follicle cells had previously been shown to exhibit characteristic patterns of membrane channels as well as membrane potential and intracellular pH. Stage-specifically, ductin and subunit a were found either colocalized or separately enriched in different regions of soma and germ-line cells. While ductin was often more prominent in plasma membranes, subunit a was more prominent in cytoplasmic and nuclear vesicles. Particularly, ductin was enriched in polar cells, stalk cells, and nurse-cell membranes, whereas subunit a was enriched in the cytoplasm of border cells, columnar follicle cells and germ-line cells. Comparably, ductin and both innexins 2 and 3 were either colocalized or separately enriched in different cellular regions. While ductin often showed a continuous membrane distribution, the distribution of both innexins was mostly punctate. Particularly, ductin was enriched in polar cells and stalk cells, whereas innexin 2 was enriched in the oolemma, and innexin 3 in centripetally migrating follicle cells. In lateral follicle-cell membranes, the three proteins were found colocalized as well as separately concentrated in presumed gap-junction plaques.

**Conclusions:**

Our results support the notion of a large variety of gap junctions existing in the *Drosophila* ovary. Moreover, since ductin is the channel-forming part of a proton pump and, like the innexins, is able to form junctional as well as non-junctional membrane channels, a plethora of cellular functions could be realized by using these proteins. The distribution and activity patterns of such membrane channels are expected to contribute to developmentally important bioelectric signals.

## Background

The concerted action of ion-transport mechanisms and gap junctions gives rise to bioelectric phenomena, like pH gradients, voltage gradients and ion fluxes within cells, tissues, organs, and even organisms. Such bioelectric phenomena are known to play regulatory roles in various developmental and regenerative processes, e.g. proliferation, differentiation, polarisation and migration [1–5].

Using a variety of approaches, we have gathered considerable information on the electrical properties as well as on the distribution and activity patterns of ion-transport mechanisms and gap junctions in the ovary of *Drosophila melanogaster* [6–19]. Following this strategy in a model system, we are trying to further clarify the roles that bioelectric phenomena play during development, e.g. for pH-regulation, osmoregulation, cell-cell communication, cell migration, cell proliferation, cell death, vitellogenesis and growth.

During the course of *Drosophila* oogenesis, characteristic extracellular current patterns [6, 20] as well as membrane-potential changes in germ-line and soma cells have been observed that partly depend on the exchange of protons, potassium ions and sodium ions [7, 21, 22]. Moreover, striking stage-specific patterns of membrane potentials and intracellular pH that characterize distinct cell populations have been described recently [19]. These bioelectric patterns were found to be related to the distribution patterns of various membrane channels, namely V-ATPases, L-type Ca^2+^-channels, amiloride-sensitive Na^+^-channels and Na^+^,H^+^-exchangers, as well as innexin-3-containing gap junctions. Therefore, these membrane proteins are likely to be involved in the regulation of membrane potentials and/or intracellular pH [19].

An ovarian follicle of *Drosophila* consists of 16 germ-line cells surrounded by a layer of somatic follicle cells [23, 24]. The oocyte and its 15 nurse cells form a cytoplasmic continuum via intercellular bridges as well as via gap junctions, and the same holds true for the follicle cells. On the other hand, the germ-line cells are connected to the soma cells only via gap junctions [25, 26]. By way of the intercellular exchange of microinjected fluorescent tracers, stage-specific communication between germ-line cells and soma cells has been detected, and a variety of treatments was found to either inhibit or stimulate this communication [12].

Innexins are considered the main gap-junction proteins of invertebrates [27–30], but there is some evidence that additional proteins, like e.g. ductin, can be part of gap junctions [11, 15, 31]. Therefore, it is tempting to analyse whether or not ductin and members of the innexin family are found in common gap-junction plaques. Previously, the localization of ductin [11, 16] as well as the localization of innexins 1 to 4 [18] have been separately analysed in *Drosophila* ovarian follicles in detail.

In the present study, we try to clarify the relationship of ductin, known as a channel-forming subunit of V-ATPases, with innexins 2 and 3, two well known components of gap junctions, using double-immunolabeling. We apply combinations of four antisera directed against (1) ductin (proteolipid or subunit c), (2) another V-ATPase component in the membrane-spanning V_0_ domain (subunit a), (3) innexin 2, and (4) innexin 3, respectively. These antisera have previously been shown to specifically recognize cellular antigens in the *Drosophila* ovary [18, 19].

## Methods

### Preparation of follicles

*Drosophila melanogaster* wild-type Oregon R flies were reared at about 20 °C on standard food with additional fresh yeast. Individual 2–3 days old females were killed by crushing the thorax with tweezers without previous anesthetization. The ovaries were dissected with tweezers in *Drosophila* PBS [32], and single follicles of different stages were isolated by pulling at the anterior tip of an ovariole.

### Antisera

To localize V-ATPase and gap-junction proteins, we used (1) a rabbit antiserum (Anti-ductin [19]) raised and affinity-purified against a highly conserved region (amino acids 115 to 126 in putative loop 3) of the 16 kDa-protein ductin, which forms subunit c of V-ATPases and is also supposed to be a part of gap junctions (AB5496, Chemicon International, USA), (2) a guinea-pig antiserum (Anti-V-ATPase a [19]) raised against an N-terminal region of subunit a of the *Manduca sexta* V-ATPase, kindly provided by B. Walz and O. Baumann (Potsdam, Germany), (3) a guinea-pig antiserum (Anti-Inx2 [18]) raised against the cytoplasmic loop of innexin 2, and (4) a guinea-pig antiserum (Anti-Inx3 [18]) raised against the C-terminus of innexin 3 from *Drosophila*, kindly provided by R. Bauer and M. Hoch (Bonn, Germany). A rabbit non-immune serum (NIS [11, 15–18]) was used as control.

### Immunoblotting

Immunoblots were performed as described previously [11]. In short, homogenates of ovaries were sonicated and briefly boiled. The proteins were separated using 12 % SDS-PAGE and transferred to nitrocellulose membranes. Nonspecific binding sites were blocked with 5 % skimmed milk powder/PBS and the blots were incubated in Anti-ductin diluted 1:100 up to 1:500 with 1 % BSA/PBS. NIS diluted 1:200 was used as a control [11]. Subsequently, biotinylated goat-anti-rabbit IgG (Jackson, PA, USA; diluted 1:1000), streptavidin-peroxidase (Dianova, Germany; diluted 1:1000) and H_2_O_2_/4-chloro-1-naphthol (Sigma, Germany) were applied, and photographs were taken using a digital camera. Experiments were performed three times.

### Indirect immunofluorescence preparations

Follicles were fixed for 30 min at 4 °C in 4 % formaldehyde dissolved in PBS, washed in PBS and blocked for 1 h at 20 °C with 2 % BSA/0,1 % Triton X-100 in PBS. Thereafter, the follicles were incubated overnight at 4 °C in PBS containing 0,5 % BSA/0,1 % Triton X-100 and two of the following antisera for double-labeling: Anti-ductin diluted 1:100, Anti-V-ATPase a diluted 1:1000, Anti-Inx2 diluted 1:20, Anti-Inx3 diluted 1:20. Controls were performed without antiserum and using NIS 1:200 [11, 18], respectively.

After washing six times for 10 min, the follicles were treated with goat-anti-rabbit-Cy3 (Jackson, USA; diluted 1:2000) and donkey-anti-guinea-pig-FP488 (FluoProbes/Interchim, France; diluted 1:100) for 1 h in PBS containing 0,5 % BSA/0,1 % Triton X-100. Washing was repeated six times and the nuclei were stained with 0,2 μg/ml DAPI (Sigma, Germany) in PBS for 3 min. Thereafter, the follicles were mounted in Fluoromount G (Interchim) and viewed, by using x20/0.5, x40/0.75 or x63/1.25oil objectives and the appropriate filter sets, either on a Zeiss Axiovert 200 wide-field fluorescence microscope (WFM), equipped with a Hamamatsu Orca ER camera, or they were mounted in glycerine/PBS 1:1 and viewed on a Leica DMRE laser-scanning microscope (LSM). Each experiment was repeated at least three times.

## Results

### Immunoblot analysis of Anti-ductin

**Fig. 1:**
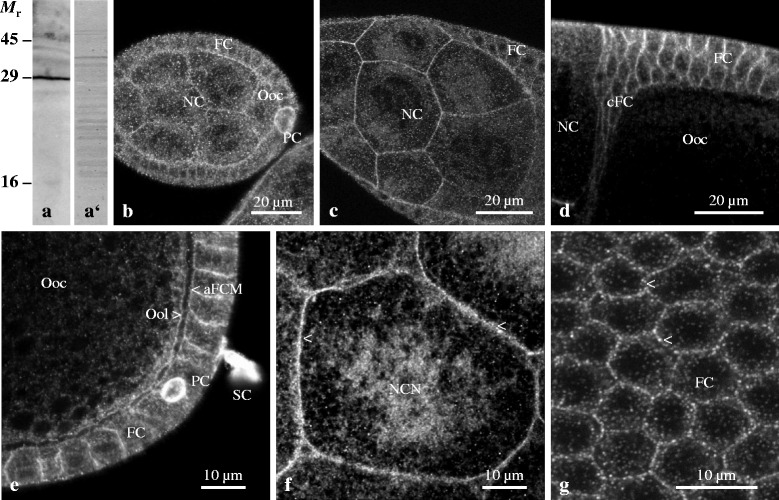
Ductin is localized stage-specifically in plasma membranes and cytoplasmic vesicles of follicle cells and germ-line cells. **a**: On immunoblots of ovary extracts using Anti-ductin, a band at 29 kDa (*M*_r_, putative dimer) and weak bands at about 16 kDa (putative monomer) and 45 kDa (putative trimer) are recognized. **a**’: No specific staining was obtained with NIS. **b** to **g**: LSM images; **b**: Ductin is found in plasma membranes and cytoplasmic particles or vesicles, especially in polar cells (PC, stage 5, optical median section). **c**: Ductin becomes increasingly localized to plasma membranes in nurse cells (NC) and follicle cells (FC, stage 9). **d**: Ductin is found in plasma membranes of centripetally migrating FC (cFC, stage 10b). **e**: Ductin is localized in lateral and apical FC membranes (aFCM), in the oolemma (Ool) and, most prominently, in PC and stalk cells (SC, stage 10b). **f**: Ductin is found in plasma membranes of squamous FC and NC (arrowheads) and around NC nuclei (NCN, stage 10b, optical tangential section). **g**: Ductin is concentrated in plaques in lateral FC membranes (arrowheads, stage 9, optical tangential section)

Using immunoblots of *Drosophila* ovary preparations, we analysed the binding properties of the commercially available antiserum AB5496 (Anti-ductin). As described earlier for two other anti-ductin sera (AN2, prepared against *Nephrops* ductin and affinity-purified against *Drosophila* ductin, and AD16, prepared and affinity-purified against a peptide specific for the N-terminus of *Drosophila* ductin (cf. [11, 15–17])), Anti-ductin recognized a strong band at 29 kDa and weak bands at 16 and 45 kDa (Fig. 1a). No specific binding was obtained with NIS (cf. [11]). The 16 kDa-band represents the monomer of ductin and the other bands represent the putative dimer and trimer, respectively. Thus, the binding of Anti-ductin in indirect immunofluorescence preparations is assumed to be specific for the channel-forming protein ductin in *Drosophila* ovarian follicles.

### Localization of ductin in indirect immunofluorescence preparations

Using Anti-ductin and laser-scanning microscopy (LSM), we investigated the distribution of ductin during the course of oogenesis (Fig. 1b-g). As shown previously using the two other anti-ductin sera on sections prepared for immuno-fluorescence and immuno-electron microscopy [11, 16], the continuous as well as punctate distribution of ductin was found to change stage-specifically. In the following, we concentrate on ductin in specialized types of follicle cells (polar cells, border cells, stalk cells and centripetally migrating cells) and on some main features of its distribution relevant to the present comparative analysis.

During previtellogenesis (up to stage 7), ductin is located in cytoplasmic particles or vesicles as well as in plasma membranes and is enriched in polar cells (Fig. 1b). Beginning with vitellogenesis, ductin increasingly becomes located in plasma membranes of nurse cells and follicle cells (Fig. 1c). During vitellogenesis (from stage 8 onward), ductin is located (1) in cytoplasmic vesicles, (2) in plasma membranes of centripetally migrating follicle cells (Fig. 1d), (3) in lateral and apical plasma membranes of columnar follicle cells, (4) in plasma membranes of squamous follicle cells and nurse cells as well as around nurse-cell nuclei, (5) in the oolemma, and (6), most prominently, in polar cells and stalk cells (Fig. 1e and f). Predominantly in lateral and apical follicle-cell membranes, ductin is localized in a punctate pattern resembling gap-junction plaques (Fig. 1g; (cf. [11])). No specific staining was found in control preparations without primary antiserum or using NIS (cf. [11, 18]).

### Colocalization of ductin with V-ATPase subunit a

**Fig. 2:**
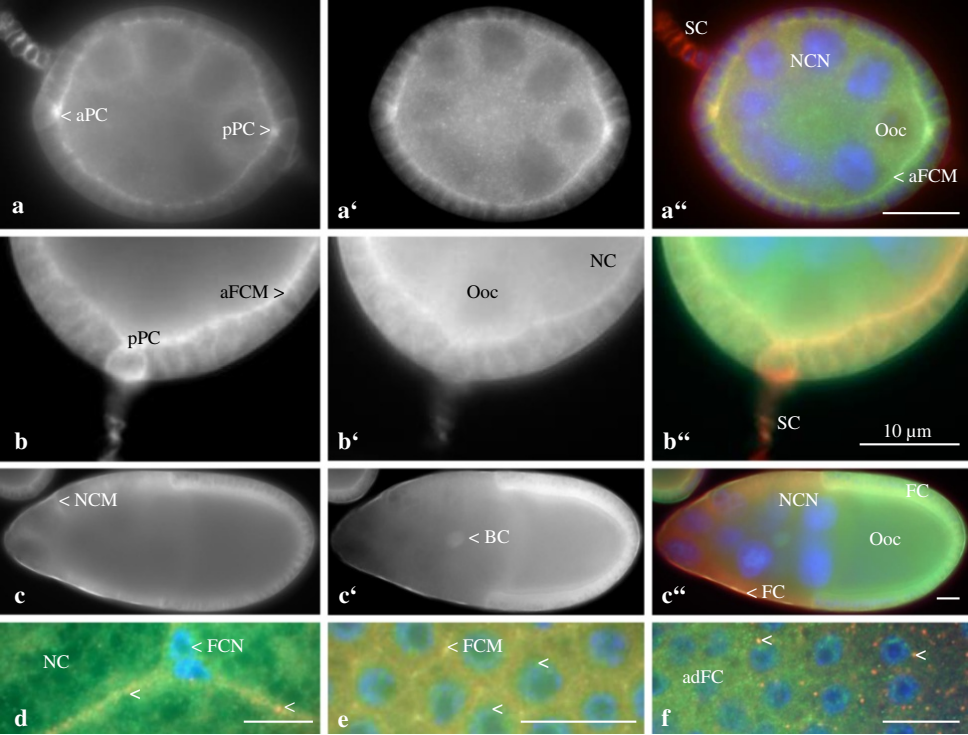
Ductin and V-ATPase subunit a are partly colocalized in plasma membranes and in the cytoplasm of follicle cells and germ-line cells, often in cytoplasmic particles or vesicles. **a** to **c**: Ductin (subunit c); **a’** to **c’**: V-ATPase subunit a; **a”** to **c”** and **d** to **f**: merged images, ductin (red), V-ATPase subunit a (green) and colocalization of both (yellow), WFM. Nuclei were stained with DAPI. No specific staining was obtained with NIS (cf. inset in Fig. 4b). In young previtellogenic follicles (**a** to **a”**, stage 4), ductin and V-ATPase subunit a co-occur in anterior and posterior polar cells (aPC, pPC), in lateral and apical follicle-cell membranes (aFCM), and in the cytoplasm of FC, nurse cells (NCN, NC nucleus) and the oocyte (Ooc). While cytoplasmic particles or vesicles containing predominantly V-ATPase subunit a can be observed (**a’**), ductin is enriched in stalk cells (SC). In older previtellogenic follicles (**b** to **b”**, stage 6), ductin and V-ATPase subunit a are enriched in aFCM. While ductin is found in higher amounts in pPC and SC, V-ATPase subunit a is found in higher amounts in the cytoplasm of FC, NC and Ooc (**b’**). In vitellogenic follicles (**c** to **c”**, stage 10a), ductin and V-ATPase subunit a are colocalized especially in columnar and squamous FC. While ductin is enriched in NC membranes (NCM), V-ATPase subunit a is enriched in border cells (BC). **d**: Ductin and V-ATPase subunit a are colocalized in particles or vesicles (arrowheads, yellow) in squamous FC (FCN, FC nucleus, stage 10b, surface view). **e**: Ductin and V-ATPase subunit a are colocalized in lateral FC membranes (FCM) as well as in the cytoplasm (yellow, stage 10b, surface view). Particles or vesicles containing V-ATPase subunit a are found in FCN (arrowheads, green). **f**: In older follicles (stage 12, surface view), ductin and V-ATPase subunit a are colocalized in particles or vesicles in most of the FC (arrowheads, yellow) while, in anterodorsal FC (adFC), predominantly V-ATPase subunit a is found in the cytoplasm and in the nuclei (green vesicles). Scale bars, 10 μm

Since ductin forms subunit c of V-ATPases, it is expected to localize, at least in part, together with subunit a in the same cellular regions. We analysed follicles double-labeled with Anti-ductin and Anti-V-ATPase a using wide-field fluorescence microscopy (WFM, Fig. 2). Stage-specifically, ductin and subunit a were found colocalized in plasma membranes and in the cytoplasm of follicle cells and germ-line cells, especially in cytoplasmic particles or vesicles.

During early previtellogenesis, ductin and subunit a co-occur (1) in polar cells, (2) in lateral and apical follicle-cell membranes and (3) in the cytoplasm of follicle cells, nurse cells and the oocyte. However, while ductin was found to be more prominent in stalk cells, the distinct particles or vesicles in germ-line cells were found to contain predominantly subunit a (Fig. 2a). During late previtellogenesis, both ductin and subunit a are enriched in apical follicle-cell membranes. On the other hand, ductin was found to be more prominent in posterior polar cells and in stalk cells, while subunit a was more prominent in the cytoplasm of follicle cells, nurse cells and the oocyte (Fig. 2b).

During vitellogenesis, ductin and subunit a co-occur especially in columnar and squamous follicle cells and in border cells. In columnar follicle cells, the amount is usually higher on one side of the follicle (Fig. 2c), namely the ventral side (cf. [19]). Here, ductin and subunit a are colocalized in the plasma membranes and in the cytoplasm while, in squamous follicle cells, they are are colocalized in vesicles (Fig. 2d). On the other hand, ductin is enriched in nurse-cell membranes, while subunit a is enriched in border cells (Fig. 2c) as well as in nuclear particles or vesicles in follicle cells (Fig. 2e). During late vitellogenesis, ductin and subunit a are colocalized in cytoplasmic vesicles in most of the follicle cells, whereas, in the cytoplasm and nuclei of anterodorsal follicle cells, predominantly subunit a was found (Fig. 2f). Taken together, ductin and subunit a are stage-specifically either colocalized or separately enriched in different cellular regions of germ-line and soma cells. Frequently, ductin is more prominent in plasma membranes, while subunit a is more prominent in cytoplasmic and nuclear particles or vesicles (for summary, see Fig. 5a and a’).

### Colocalization of ductin with innexins

Since ductin is known to form intercellular communication channels [11, 15, 31], it might localize, in part, together with members of the channel-forming innexin family in the same cellular regions [27–30]. In *Drosophila* ovarian follicles, the localization of innexins 1 to 4 has been described in detail previously using LSM and WFM [18]. For the present comparative analysis, we concentrate on some main features concerning Inx2 and Inx3, that both showed distribution patterns similar to ductin.

### Ductin and Inx2

**Fig. 3:**
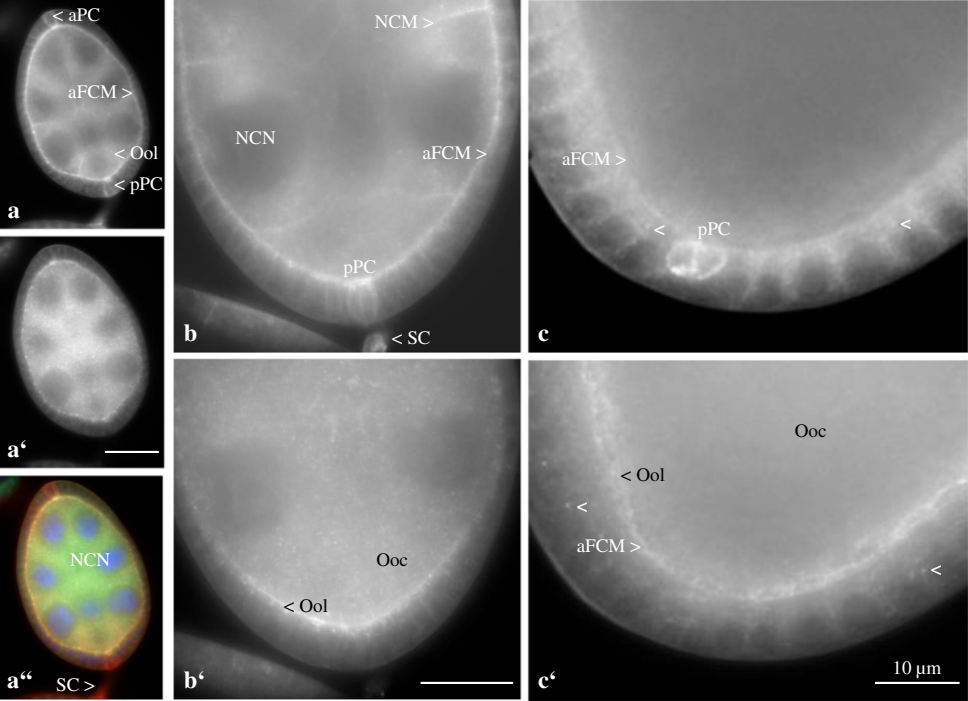
Ductin and Inx2 are partly colocalized in plasma membranes and in the cytoplasm of follicle cells and germ-line cells, but often in different plaques. **a** to **c**: Ductin; a’ to c’: Inx2; A”: merged image, ductin (red), Inx2 (green) and colocalization of both (yellow), WFM. Nuclei were stained with DAPI. No specific staining was obtained with NIS (cf. inset in Fig. 4b). In previtellogenic follicles (**a** to **a”**, stage 6; **b** to **b’**, stage 7), ductin and Inx2 co-occur in lateral and apical follicle-cell membranes (aFCM) and in the cytoplasm of FC, nurse cells (NCN, NC nucleus) and the oocyte (Ooc). While prominent cytoplasmic particles or vesicles containing Inx2 can be observed in germ-line cells (**a’**, **b’**), ductin is found to be enriched in NC membranes (NCM), in the oolemma (Ool), in anterior and posterior polar cells (aPC, pPC) and in stalk cells (SC). In vitellogenic follicles (**c** to **c’**, stage 10b), ductin and Inx2 co-occur in lateral and apical FC membranes (FCM), while ductin is found to be enriched in pPC, and Inx2 in the Ool, respectively. Both proteins are concentrated in lateral FCM, but they often localize in different plaques (cf. arrowheads in **c** and **c’**). Scale bars, 10 μm

We analysed follicles double-labeled with Anti-ductin and Anti-Inx2 using WFM (Fig. 3). During previtellogenesis (Fig. 3a, b), ductin and Inx2 co-occur (1) in lateral and apical follicle-cell membranes and (2) in the cytoplasm of follicle cells, nurse cells and the oocyte. However, ductin was found to be enriched (1) in nurse-cell membranes and (2) in the oolemma as well as (3) in polar cells and (4) in stalk cells, whereas cytoplasmic particles or vesicles containing predominantly Inx2 were observed in germ-line cells.

Also during vitellogenesis (Fig. 3c), ductin and Inx2 co-occur in lateral and apical follicle-cell membranes. On the other hand, ductin is still enriched in posterior polar cells, while Inx2 is now enriched in the oolemma. In lateral follicle-cell membranes, both proteins are often found concentrated in different plaques that are presumed to represent different gap junctions (arrowheads in Fig. 3c and c’).

### Ductin and Inx3

**Fig. 4:**
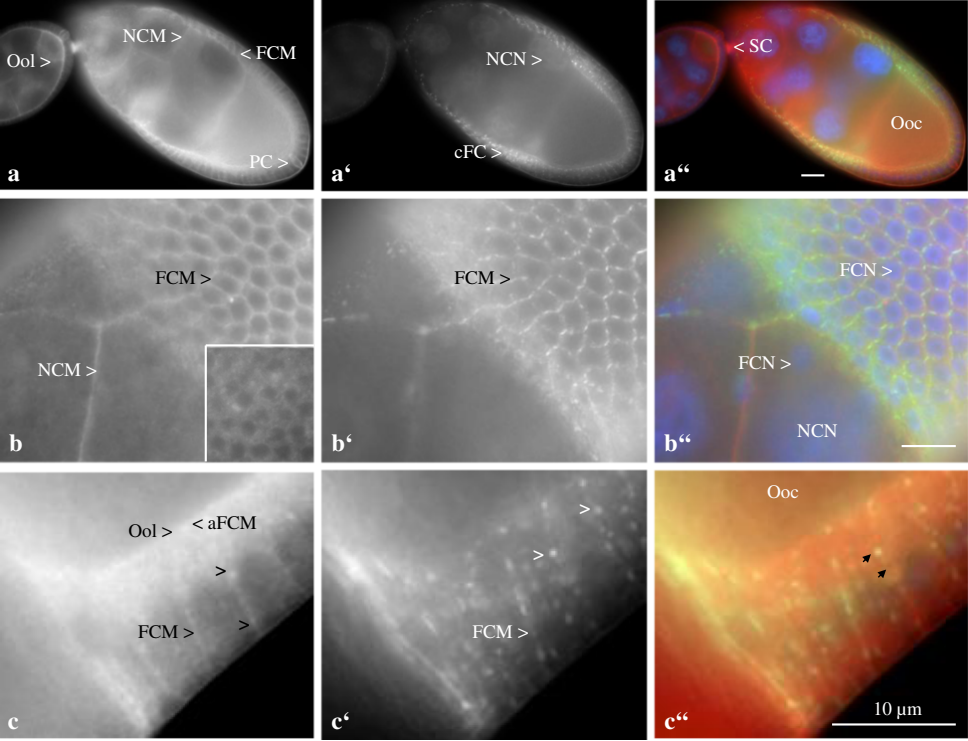
Ductin and Inx3 are partly colocalized in plasma membranes of follicle cells and germ-line cells, but often in different plaques. **a** to **c**: Ductin; **a’** to **c’**: Inx3; **a”** to **c”**: merged images, ductin (red), Inx3 (green) and colocalization of both (yellow), WFM. Nuclei were stained with DAPI. No specific staining was obtained with NIS (inset in **b**). In previtellogenic and early vitellogenic follicles (**a** to **a”**, stage 6, left, and stage 9, right), ductin and Inx3 co-occur in follicle-cell membranes (FCM). While ductin is found to be enriched in nurse-cell membranes (NCM), in the oolemma (Ool), in polar cells (PC), in stalk cells (SC) and in the cytoplasm of NC and oocyte (Ooc), Inx3 is enriched especially in lateral membranes of the prospective centripetally migrating FC (cFC) and around NC nuclei (NCN). In later stages (**b** to **b”**, surface view, and **c** to **c”**, optical median section, stage 10b), ductin and Inx3 co-occur in lateral FCM (FCN, FC nucleus) and in NCM. While Inx3 exhibits prominent membrane plaques (**b’**), ductin is enriched in apical FCM (aFCM) and in the Ool **c**. Both proteins are concentrated in lateral FCM, but they often localize in different plaques (cf. arrowheads in **c** and **c’** and arrows in **c”**). Scale bars, 10 μm

Follicles double-labeled with Anti-ductin and Anti-Inx3 were also analysed using WFM (Fig. 4). In previtellogenic and early vitellogenic stages (Fig. 4a), ductin and Inx3 co-occur in follicle-cell membranes. However, ductin is more prominent (1) in nurse-cell membranes and (2) in the oolemma as well as (3) in polar cells, (4) in stalk cells and (5) in the cytoplasm of germ-line cells. On the other hand, Inx3 was found to be enriched around nurse-cell nuclei and in lateral plasma membranes of the prospective centripetally migrating follicle cells.

In mid to late vitellogenic stages (Fig. 4b, c), ductin and Inx3 co-occur in lateral follicle-cell membranes and in nurse-cell membranes. While ductin is enriched in apical follicle-cell membranes and in the oolemma, Inx3 forms prominent plaques in lateral follicle-cell membranes. Here, ductin and Inx3 are often found concentrated in different plaques that are presumed to represent different gap junctions (arrowheads in Fig. 4c and c’ and arrows in c”).

**Fig. 5:**
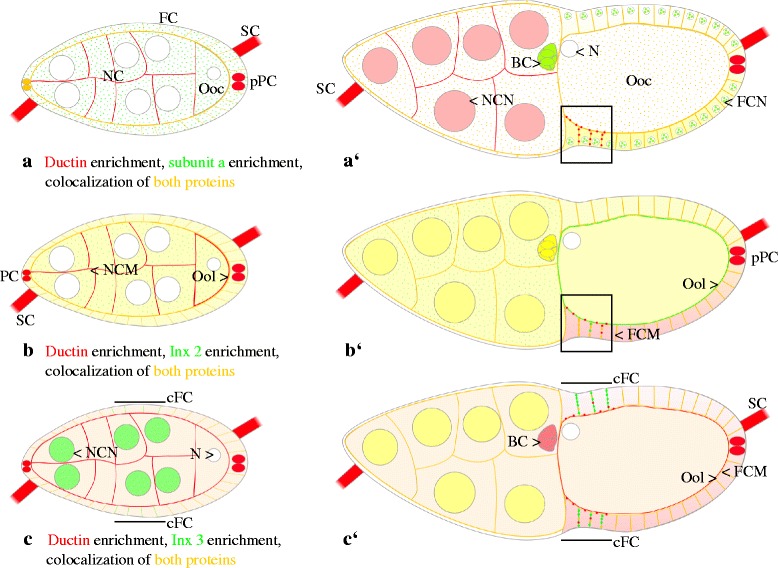
Summary of colocalization or local enrichment of ductin, V-ATPase subunit a, Inx2 and Inx3, respectively, during previtellogenic stages (up to stage 7) and vitellogenic stages (up to stage 13). **a**, **a’**: Ductin (subunit c) and V-ATPase subunit a; **b**, **b’**: Ductin and Inx2; **c**, **c’**: Ductin and Inx3. Ductin and subunit a (**a**, **a’**) are stage-specifically either colocalized in plasma membranes or in the cytoplasm (yellow), which is supposed to represent the distribution of V-ATPases, or separately enriched in different cellular regions of germ-line and soma cells. Frequently, ductin (red) is more prominent in plasma membranes, while subunit a (green) is more prominent in cytoplasmic and nuclear particles or vesicles. Punctate membrane labeling (red, example shown in the box) is presumed to originate from ductin as part of membrane channels or gap-junctional structures. **a**: In previtellogenic stages (e.g. stage 7 is shown), ductin (red) is enriched in posterior polar cells (pPC) and in stalk cells (SC), while subunit a (green) is enriched in cytoplasmic particles or vesicles in follicle cells (FC), nurse cells (NC) and the oocyte (Ooc). **a’**: In vitellogenic stages (e.g. stage 10b is shown), the dorsoventral gradient of V-ATPase distribution in the FC epithelium (yellow) is in accordance with the gradual distribution of epithelial membrane potentials [19]. While ductin (red) is enriched in NC, pPC and SC, subunit a (green) is enriched in border cells (BC) and nuclear particles or vesicles in FC (FCN; N, oocyte nucleus; NCN, nurse-cell nuclei). Since PC, BC and ventral FC are also characterized through a high activity of L-type Ca^2+^-channels, alterations of membrane potentials through V-ATPase activity are supposed to result in cellular responses due to voltage-dependent Ca^2+^-currents [19]. Ductin and Inx2 (**b**, **b’**) as well as ductin and Inx3 (**c**, **c’**) are stage-specifically either colocalized (yellow) or separately enriched (red vs. green) in different cellular regions of germ-line and soma cells. Frequently, ductin (red) shows a rather continuous membrane distribution, presumably representing V-ATPases, while the distribution of both innexins (green) is more punctate, presumably representing gap-junctional structures. In lateral FC membranes (FCM), both ductin and Inx2 as well as ductin and Inx3 are often found concentrated in different plaques (red vs. green) that are presumed to represent different gap-junctional structures (example shown in the box in **b’**). **b**: In previtellogenic stages, ductin (red) is enriched in NC membranes (NCM) and in the oolemma (Ool) as well as in PC and SC, whereas Inx2 (green) is enriched in cytoplasmic particles or vesicles in germ-line cells. **b’**: In vitellogenic stages, ductin (red) is enriched in pPC, while Inx2 (green) is enriched in the Ool. Antibodies directed against presumed cytoplasmic regions of both ductin or Inx2 microinjected into the oocyte specifically blocked intercellular communication through ductin- or Inx2-containing gap junctions [11, 15, 18]. **c**: In previtellogenic stages, ductin (red) is more prominent in NCM and in the Ool as well as in PC, in SC and in the cytoplasm of germ-line cells, while Inx3 (green) is more prominent around NCN and in lateral plasma membranes of the prospective centripetally migrating FC (cFC, bars). **c’**: In vitellogenic stages, ductin (red) is enriched in apical FCM and in the Ool as well as in pPC, BC and SC, while Inx3 (green) forms prominent plaques in lateral membranes of cFC (bars). Inx3-containing gap junctions are supposed to specifically regulate the distribution of membrane potentials, intracellular pH and ions within cFC and to establish a communication border to neighboring FC [12, 13, 16, 19, 60]

Taken together, ductin and both innexins are stage-specifically either colocalized or separately enriched in different cellular regions of germ-line and soma cells. Frequently, ductin shows a rather continuous membrane distribution, while the distribution of both innexins is more punctate (for summary, see Fig. 5b, b’ and c, c’).

## Discussion

The channel-forming protein ductin has been found in both V-ATPases and gap-junctional structures [31]. In V-ATPases, it forms the proteolipid component or subunit c, whereof the C- and N-termini as well as loop 2 are located either on the vacuolar or on the extracellular side of the membrane. In gap-junctional structures, where ductin is supposed to form membrane channels, these regions are located on the cytoplasmic side of the membrane [33, 34]. Using light and electron microscopy, it has been shown that antibodies against ductin bind to antigens located in the cytoplasm and in plasma membranes of various *Drosophila* tissues, especially of ovarian follicles and embryos [11, 15–17]. In part, cytoplasmic labeling might represent storage or transport of ductin. However, since V-ATPases are required for the acidification of organelles (e.g. [35]), cytoplasmic labeling is likely to also represent secretory vesicles, lysosomes or endosomes in which ligand-receptor complexes become dissociated (e.g. [16, 36]).

On the other hand, V-ATPases coupled to secondary active antiport mechanisms are known to energize transport processes across plasma membranes (e.g. [37]). These pumps are expected to exert direct or indirect influence on intracellular pH and membrane potentials. In the follicle-cell epithelium of *Drosophila*, antibodies against both subunits a and c revealed an asymmetric distribution of V-ATPases which is in accordance with the gradual dorsoventral distribution of epithelial membrane potentials [19]. This observation points to a possible involvement of V-ATPases in the regulation or maintainance of the spatial coordinates during oogenesis, a phenomenon that has been observed in other systems (e.g. [38–40]). Moreover, V-ATPases are also known to be involved in the regulation of cell division, migration and differentiation (e.g. [41, 42]).

Labeling of plasma membranes with antibodies against ductin was either continuous or punctate (cf. [11, 16, 19]). Continuous labeling is presumed to originate from ductin as part of V-ATPases, while punctate labeling might originate from ductin as part of gap-junctional structures. By microinjection experiments, it has been shown that antibodies directed against presumed cytoplasmic regions of ductin specifically block intercellular communication and exert adverse influence on oogenesis as well as on embryogenesis [11, 15].

Interestingly, ductin labeling was prominent in both stalk cells and polar cells that derive from a common cell population in the germarium. Polar cells are known to control stalk formation as well as axis formation, they recruit the border cells and they express various cell-specific markers [43, 44].

Evidence amounts that ductin, or subunit c, is a multi-functional channel protein. It has been observed to form Ca^2+^-inducible membrane pores that are permeable to hydrophilic molecules [45]. It has also been demonstrated that hexamers of subunit c form stacked rings containing an intercellular channel [46, 47]. And, in addition, ductin seems to play a role in the process of vesicle-membrane fusion [48]. The present study lends further support to the notion that ductin might be engaged in several of these functions also during *Drosophila* oogenesis.

Although various proteins have been described to be part of *Drosophila* gap junctions (cf. [15, 27]), members of the innexin family are assumed to be the main invertebrate gap-junction proteins [29, 30, 49]. In the *Drosophila* ovary, the mRNAs of innexins 1, 2, 3, 4 and (to a minor extent) 7 have been detected [50]. Using various antibodies against innexins (Inx), Inx1 was found to be predominantly located in the baso-lateral domain of follicle cells, whereas Inx2 is positioned apico-laterally as well as apically between follicle cells and germ-line cells. Inx3 was observed laterally in follicle cells and also in nurse cells, and Inx4 was detected in the oolemma up to stage 8 and in nurse-cell membranes up to stage 12. While Inx2 and Inx3 are colocalized between somatic cells, Inx2 and Inx4 are colocalized between somatic cells and germ-line cells [18].

Microinjected antibodies directed against presumed cytoplasmic regions of Inx2 specifically blocked intercellular communication and lead to inhibitory effects on oogenesis that were similar to those obtained with antibodies directed against presumed cytoplasmic regions of ductin [18]. Even during spermatogenesis, Inx2 was found to be involved in soma-to-germ line intercellular communication [51]. Moreover, it has been demonstrated that, within the germarium, Inx2 supports germ-cell survival, cyst formation and early follicle development [52].

During embryogenesis, Inx2 was shown to participate in organizing epithelia by interacting with core proteins of adherens and septate junctions, and it has been presumed that Inx2 exerts its structural functions indirectly through the formation of channels for intercellular communication [53–56]. Recently, Inx2 has also been identified as a regulator of eye development [57].

Contrary to Inx2, Inx3 shows a striking non-uniform distribution in the follicle-cell epithelium [19]: it is enriched in the lateral membranes of centripetally migrating follicle cells. This indicates distinct coupling conditions between these cells concerning e.g. regulatory signals, membrane potentials or intracellular pH. Moreover, Inx3 has been described to be involved in maintaining tissue integrity in response to tension [58], a feature that is particularly important for migrating cells. The spatial restriction of specialized intercellular channels and the independent regulation of their permeability is suspected to provide mechanisms to generate or maintain boundaries between distinct follicle-cell populations [19]. By the use of various proteins in either homomeric, heteromeric or heterotypic combinations, a wealth of different gap-junction channels might be formed (cf. [18]).

Compared to neighboring cells, the centripetally migrating follicle cells are specialized in several respect: (1) they are more acidic and more depolarized [19], (2) they possess higher amounts of active voltage-dependent L-type Ca^2+^-channels [19] (becoming activated under depolarized conditions [59]), (3) they contain higher Ca^2+^-concentrations [60], (4) they possess higher amounts of Na^+^,K^+^-ATPase [13, 16] (becoming stimulated under alkaline conditions [10]), and (5) they remain coupled via gap junctions to the oocyte when neighboring cells are already uncoupled [12].

The permeability of innexin channels in general has been reported to be sensitive to membrane potential, intracellular pH, K^+^- and Ca^2+^-concentrations (e.g. [61, 62]). Gap-junctional communication between oocyte and follicle cells was found to be inhibited by low extracellular pH, by low extracellular K^+^-concentrations (leading to hyperpolarization [21, 22]), and by high Ca^2+^-concentrations in the oocyte [12]. Taken together, these findings support the notion that intercellular communication in *Drosophila* ovarian follicles is precisely regulated.

Similar to ductin and to the pannexins in vertebrates, innexins have also been found to form non-junctional channels (innexons) allowing the secretion of small molecules, including ATP, into the extracellular space [47, 62–64]. Innexons were observed to open in response to mechanical stress, depolarization, high intracellular Ca^2+^- and high extracellular K^+^-concentrations, and to close due to low intracellular pH [62, 63].

## Conclusions

In recent years, the origin as well as the physiological and cellular functions of bioelectric phenomena have attracted growing attention and, thus, their meaning becomes more and more elucidated (e.g. [4, 65–68]). Accordingly, the distribution and activity patterns of membrane channels, like ductin and innexins, during the course of *Drosophila* oogenesis are supposed to contribute to developmentally important bioelectric signals [19].

Since ductin shows overlapping distribution patterns with both Inx2 and Inx3 (for summary, see Fig. 5), these proteins might be, in part, located within the same gap-junctional structures, especially between neighboring follicle cells. However, the analysed proteins are often found concentrated in different membrane plaques which supports the notion of different gap junctions with a large variety of protein compositions.

Particularly, ductin is enriched in polar cells and stalk cells, while Inx2 is enriched in the oolemma, and Inx3 is enriched in centripetally migrating follicle cells, but the other proteins are not absent from these regions. Since ductin is part of a proton pump and, like the innexins, can form junctional as well as non-junctional membrane channels, a plethora of cellular functions could be realized by using only the three proteins analysed in the present study.

## Abbreviations

a, anterior; ad, anterodorsal; BC, border cells; BSA, bovine serum albumine; c, centripetally migrating; DAPI, 4’,6-diamidino-2-phenylindole; DMSO, dimethyl sulfoxide; FC, follicle cells; Inx2, innexin 2; Inx3, innexin 3; LSM, laser-scanning microscopy; M, membrane; N, nucleus; NC, nurse cells; NIS, non-immune serum; Ooc, oocyte; Ool, oolemma; p, posterior; PBS, phosphate buffered saline; PC, polar cells; SC, stalk cells; SDS-PAGE, sodium dodecyl sulphate polyacrylamide gel-electrophoresis; WFM, wide-field microscopy
